# Italian Consumers’ Readiness to Adopt Eggs from Insect-Fed Hens

**DOI:** 10.3390/ani11113278

**Published:** 2021-11-16

**Authors:** Nico Lippi, Stefano Predieri, Camilla Chieco, Giulia Maria Daniele, Marta Cianciabella, Massimiliano Magli, Lara Maistrello, Edoardo Gatti

**Affiliations:** 1 Institute of BioEconomy (IBE), National Research Council, Via P. Gobetti, 101, 40129 Bologna, Italy; nico.lippi@ibe.cnr.it (N.L.); camilla.chieco@ibe.cnr.it (C.C.); giuliamaria.daniele@ibe.cnr.it (G.M.D.); marta.cianciabella@ibe.cnr.it (M.C.); massimiliano.magli@ibe.cnr.it (M.M.); edoardo.gatti@ibe.cnr.it (E.G.); 2 Centre BIOGEST-SITEIA, Department of Life Science, Università di Modena e Reggio Emilia, Via G. Amendola, 2, 42122 Reggio Emilia, Italy; lara.maistrello@unimore.it

**Keywords:** insects, eggs, laying hens, insects as feed, consumers’ inclination, neophobia

## Abstract

**Simple Summary:**

An overall increase in food demand is pushing the agri-food sector toward higher food output. In particular, the feeding of laying hens plays a major role, requiring larger quantities of soybean meal for egg production each year. Replacing soybean meal with insect meal has proven to lower the environmental impact compared to traditional feed production, but it may influence consumer acceptance. An online survey was conducted to evaluate the perception and the readiness of Italian consumers to the use of eggs from laying hens fed with insect meal. The results showed a considerable level of willingness of the participants to face this innovative scenario.

**Abstract:**

Demand for eggs from laying hens is increasing as the world population continues to grow. The use of insects as animal feed is a strategic opportunity to find a new innovative, economic and sustainable source of protein. The aim of this study was to evaluate the Italian consumer inclination for eggs produced with the use of insect-fed hens. The investigation on consumers’ readiness to adopt eggs from insect-fed hens was carried out through an online survey conducted on 510 participants. Survey results outlined four different clusters on the basis of the willingness to buy/eat eggs fed with insects: “ready” (40.1%), “environmentalist” (24.3%), “cautious” (21.7%), and “reluctant” (13.9%). “Ready”, “environmentalist”, and “cautious” were quite favorable to the use of insects as feed stuffs and share drivers for product choice: cheap, organic, and with an explicit indication of use of insects. On the other hand, for the “reluctant”, the diet based on insects was the main negative factor influencing product acceptance. This cluster also showed the highest level of neophobia. Italian consumers showed a considerable level of readiness to accept insects as feed material for egg production, which should be reinforced with further information on the origin and the environmental benefit of using insects.

## 1. Introduction

The growing demand for food, due to the constant increase in the world population, which is estimated to reach 9.6 billion people in 2050 [1], is pushing the entire agri-food sector to continuously intensify production, especially for foodstuffs of animal origin. Livestock feeding for the European Union (EU) results to be a major issue, with import rates accounting for about 70% of total animal feed [2] and, from an economic point of view, animal feeding accounts for about 50–70% of the total cost of livestock production [3]. On the other hand, along the global food supply chain, each year, about one-third of raw materials that still have nutritional value is lost as leftover [4]. Nowadays, those two aspects seem to be increasingly linked to each other, especially when focusing on the circular economy scenario based on waste reduction and recycling [5]. To avoid waste along the food chain, a large number of by-products can be re-used as livestock feed, exploiting their residual nutritive value. Furthermore, by-products can be recycled as an innovative source of protein for feedstuffs [5,6,7].

An effective approach comes from researches on the use of insects as a sustainable source of protein in livestock feed. Some insects can grow in a low-value substrate, such as by-product or organic residues from the agri-food sector, which, instead of being disposed of or incinerated, can be used to produce new livestock feed by applying the concept of circular economy [8,9,10]. Moreover, insect farming is more sustainable in terms of water consumption and land exploitation than the most used crops for livestock feeding [11,12]. In addition, the residual biomass where insects are grown turns out to be rich in organic compounds and could be used as fertilizer for new crops [13,14,15].

The constant increase in protein requirements also affects the egg sector. Eggs are considered an accessible source of protein and nutrients worldwide, with annual demand of around 75 million tons expected to grow, especially in countries such as China, India, Latin America, and parts of Africa [16]. This factor has been paralleled by an increased soybean production to afford feed for laying hen diet. A significant further increase in soybean production will not be possible in the future because this crop requires large arable land and water supplies; moreover, the soybean used in animal feed is in direct competition with the same product for human consumption [17]. A number of studies have investigated alternative feeding sources for laying hens, including some that approach the concept of insect-based feed [18,19]. The main qualitative differences between eggs produced through insect-based feeds and eggs produced through regular feeds were in terms of shell thickness and yolk color [20,21]. Further relevant attributes of eggs are the impact of rearing on animal welfare as well as the environmental impact [22], which may affect the final price and are related to the consumer willingness to buy eggs. Although there is a growing awareness of nutritional and environmental benefits of adopting insect food, Western consumers, compared with Eastern ones, exhibit negative evaluations and low readiness [23,24]. An important issue affecting acceptance is the insect perception, often considered disgusting or disease vectors instead of valuable food or feed [25]. This perception is linked to food neophobia, the tendency to reject new or unfamiliar food [26]. Consumer inclination for novel foods or given food habits are also approached as readiness [27], which is an important requirement for understanding market opportunities. The aim of this study, which was conducted within the Flies4Value project, was to investigate the readiness of Italian consumers to adopt eggs from insect-fed hens (IFH) in their diet. The work, conducted through an online survey on Italian consumers, defines four clusters of acceptability of IFH. The clusters were described as related to neophobia and consumption of food categories. Conjoint analysis was used to investigate the relative importance of choice attributes within the clusters and to better understand the readiness of Italian consumers to accept insects as feed material for egg production.

## 2. Materials and Methods

### 2.1. Study Design

This research study employed a quantitative approach in the form of an online cross-sectional survey on consumer acceptance and inclination to consume eggs deriving from insect-fed hens. Inclusion criteria for the study were that participants had to be over 18 years old and currently residing in Italy. A questionnaire designed using Google form was accessible online at the Food Quality Research Group website (https://www.gustosalutequalita.it, last accessed on 14 September 2020) during June and July 2020. Participants were recruited on a national basis through advertisements posted on websites and social networks. Participation in the research was voluntary, and the right to privacy and data protection was respected in accordance with current legislation (GDPR 2016/679).

### 2.2. Questionnaire

The questionnaire was developed for the purpose of this study, based on a review of existing methods that have been used to assess consumer acceptance of novel foods [28]. Willingness to buy and consume eggs from IFH was investigated with the aim of segmenting consumers, through a single direct question, “Would you buy and eat eggs laid by hens fed with insect-derived meal?”, with four possible answers: (1) yes, hens already eat insects as their natural diet (“ready”); (2) yes, if it will help reduce environmental impact (“environmentalist”); (3) only if they come from a controlled production chain (“cautious”); and (4) never or no, because there is not yet enough information on insects used as feed (“reluctant”). Additional information required included socio-demographic features: age, sex, education, place of residence, income level. The degree of acceptance of the use of insects as food for livestock feeding was assessed using a seven-point Likert scale (1 = definitely no; 7 = definitely yes). Food neophobic attitude was quantified using the Food Neophobia Scale (FNS) developed [29] and validated in Italian by Laureati et al. [30]. The FNS consists of ten items assessed with a 7-point agreement scale ranging from 1 = “totally disagree” to 7 = “totally agree”. Multiple-choice grids were used to investigate eating habits and egg quality variables. Regarding eating habits, different types of food (red meat, white meat, dairy products, eggs, fish, dried fruit, fresh fruit, vegetables, and cereals) were listed in the column, while different time choices in terms of consumption (1: never, 2: once a week, 3: twice a week, 4: more than twice a week, 5: every day) were listed in the rows. As for egg quality variables, eggs attributes (dimension, shell color, shell integrity, kind of rearing, yolk color, type of hen feed used, brand, organic production, origin, nutrition facts label) were listed in the column, while in the rows a seven-point Likert scales (1 = not important at all; 7 = very important) allowed the evaluation of each attribute.

#### Conjoint Analyses

Conjoint analysis was conducted presenting full product profiles to consumers’ evaluations in a rating task using a full factorial design that included all possible combinations of attribute levels. Attributes and levels included in the conjoint design were chosen based on a review of the scientific literature on consumers’ preferences of eggs and based on national and European regulation for this specific product. The conjoint design consisted of three attributes: price (3 levels: 1.55 €, 1.85 €, 2.50 €), rearing type (3 levels: free-range, barn, organic rearing) and an indication on the use of insect meal as feed (2 levels: yes, or not), for a total of 18 combinations (Table 1). With reference to the first attribute, six-egg pack prices were defined in line with those indicated by the ISMEA (Italian Institute of Services for the Agricultural and Food market) report 2018 and published in March 2019 [31]. Regarding the second attribute, three different levels of rearing conditions were chosen among those indicated by Italian regulation on egg production, on the basis of the Commission Regulation (EEC) No 1274/91 and 2092/91 certain marketing standards for eggs [32,33]. Moreover, it is widely recognized that this attribute is one of the most important aspects of consumers’ purchase decisions [34]. In the current investigation, conjoint analysis is used to understand how indications on the use of insect meal as feed influence consumers preferences of eggs. For our study, we selected as the third attribute two levels of feed given to chickens based on the use of insects.

**Figure 1:**
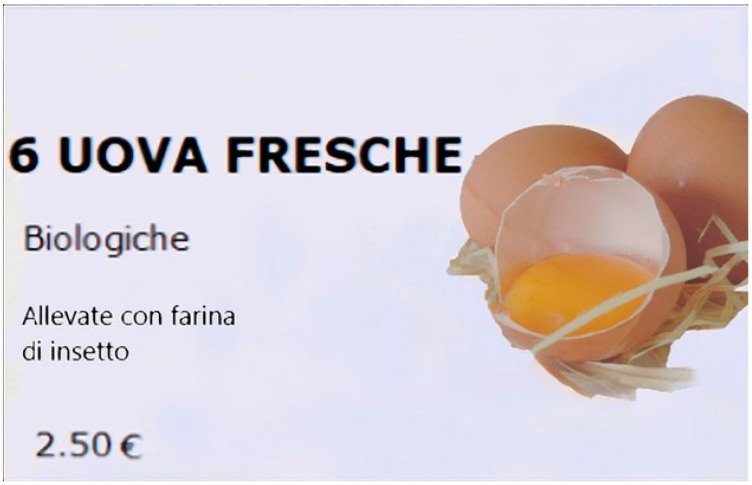
Example of a card presented in the online survey.

The test in a holistic approach was conducted by means of 18 cards, each one representing a six-pack of eggs in a pic torial presentation with attribute indications (Figure 1). Cards were presented in a monadic sequential presentation balanced for order and carry-over effects. Respondents were asked to rate the relative convenience of each card using a seven-point Likert scale (1 = extremely negative; 7 = extremely positive).

The product is described (“*uova fresche*” = fresh eggs), and the attribute level (rearing system: “*biologiche*” = organic; use of insects as a feed: “*allevate con farina di insetto*” = diet based on insect meal; price: 2.5 euro) is indicated.

### 2.3. Data Analysis

Data were analyzed with IBM^®^ SPSS^®^ release 26. Statistical differences of means were assessed according to Duncan’s multiple range test. Frequency counts for each of the terms related to individual sustainable food choices were determined by counting the number of consumers that used that term. Cochran’s Q test [35] was carried out to identify significant differences among clusters for each of the terms (*p* < 0.10).

For the conjoint analysis, part-worth utilities were estimated using Ordinary Least Squares regression analysis. This is the most extensively used method and allows to establish the relative importance of the attributes and the part-worth of each of their levels.

## 3. Results

### 3.1. Total Distribution

The total number of respondents was 510. The description and distribution of the sample in terms of socio-demographic features involved sex, education background, place of residence, income level, and age (Table 2).

### 3.2. Willingness to Consume IFH Eggs

Respondents recorded an average FNS of 29.5 and an average acceptance score for eggs from laying hens with a diet based on insects of 5.5. The core of the survey was the question on the willingness to buy and eat IFH eggs. According to answers to that multiple-choice question, respondents were grouped into four clusters characterized by decreasing levels of inclination for IFH eggs consumption: “ready”, “environmentalist”, “cautious”, and “reluctant”. The “Ready” cluster, represented by 40.1% of the respondents, accepts IFH eggs because they agree with the statement that hens are used to eating insects in their natural diet. “Environmentalists” (24.1%) are in favor of IFH eggs because of a reduced environmental impact. Consumers in the “cautious” cluster (21.7%) ask for more information before deciding. The “reluctant” cluster, which does not accept IFH eggs, was represented by 13.9% of the respondents (Table 3). FNS calculated on the whole population was found to be negatively correlated with the acceptance of insects as feed (−0.354 *p* ≤ 0.01) and showed a significant increase with age (correlation FNS-Age: 0.138 *p* ≤ 0.01). Differences due to neophobia were also observed among clusters, with the “reluctant” showing the highest level of FNS. Acceptance of general use of insects as feed, not only for hens, discriminated the “ready” and the “environmentalists” from the other two clusters, with the “cautious” recording a higher acceptance score than the “reluctant” (Table 4).

### 3.3. Eating Habits

The results showed the most relevant frequencies for fruit and vegetable consumption (Table 5) for the whole population. On the other hand, significant differences were observed among clusters for meat, with the “environmentalists” recording the lowest frequency, while “cautious” and “reluctant” recorded lower consumption of plant-derived products, legumes, and vegetables, respectively.

### 3.4. Sustainable Food Choice

**Figure 2:**
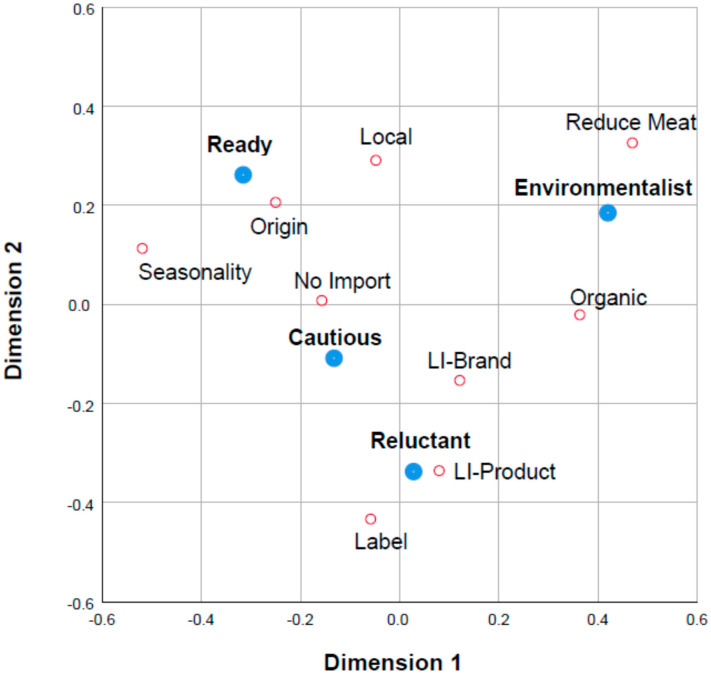
Correspondence analysis of individual sustainable food choices for the consumer clusters “ready”, “environmentalist”, “cautious”, “reluctant”. Indicators: “I choose low-impact food products” = LI-product; “I choose according to the origin of the food product” = Origin; “I choose brands with low environmental impact production” = LI-Brand; “I always prefer to buy organic food” = Organic; “I read the product label carefully” = Label; “I prefer to buy local products” = Local; “I buy seasonal products” = Seasonality; “I avoid foreign brands” = No import, “I’ve reduced my meat consumption” = Reduce Meat. N.B. The quotes for “I haven’t changed my eating habits” were not considered since they accounted for less than 5% of the respondents.

Frequency counts for each of the terms related to individual sustainable food choices were tabulated across consumer clusters and converted to a percentage. These data were organized into a consumer cluster × sustainable food choices matrix containing the percentage of consumers who checked each term related to sustainable food choices (Table 6). Among individual food choices driven by environmental issues, those related to seasonality and origin were the most frequently cited by all consumers (83.9% and 72.2%, respectively), with significant differences among the clusters for origin. The reduction in meat (44.7%) and the preference for organic foodstuffs (23.1%) were significantly higher in the “environmentalist” cluster than in the other three clusters (average 58.5% and 30.9%, respectively). Correspondence analysis shows that “environmentalists” are associated with meat reduction and organic food choice habits. “Ready” and “cautious” are concerned with seasonality, origin, and local food, the latter being more associated with a rejection of imported products. For the “reluctant” cluster, sustainable food choices are based on label claims and trust in low-impact products and brands (Figure 2).

### 3.5. Egg Attributes That Guide Consumers’ Choices

With regard to the attributes guiding the choice of eggs, the most important factors were those associated with production features (rearing, feeding, origin), with product features, such as shell color end egg size, being less relevant. Comparing the consumer clusters (Table 7), the “reluctant” were the most interested in yolk color and brand, and the “cautious” were also interested in origin. The “ready” cluster showed the lowest interest in most parameters, except for rearing, that recorded similar interest from all clusters.

### 3.6. Relative Importance of the Commercial Attributes and Part-Worth Utilities

The conjoint analysis indicated that price was the attribute with the highest relative importance with 41% of the total. The cheapest option (1.55 €) had the highest positive driver value (0.380), this parameter being inversely proportional to price. The second attribute, in terms of relative importance, was the rearing system with 33%. The free-range system was the least appreciated driver, while organic rearing resulted positively (Table 8). The last attribute, in terms of relative importance, was the use of insects as feeding material, with 25% of the total importance, with the presence of insect feed in the diet of hens having a positive orientation. The total sample scenario was respected within the clusters, with the exception of the “reluctant” cluster where the highest relative importance was represented by the attribute ‘presence of insects’, with 48% of the total importance. Furthermore, for the “reluctant” cluster, the second most significant attribute, in terms of importance, was the rearing system (31%), with the main positive driver associated with the free-range system, while the organic system was evaluated negatively. Regarding price, the “reluctant” cluster was the least influenced by the price, with 21% of the total importance and with the average price option (1.85 €) being the main negative driver.

## 4. Discussion

To support the introduction of an innovative food product into the market is appropriate to investigate its overall acceptance but also segment consumers according to issues including eating behaviors, market preferences, and ethics-based choices. Questionnaire results indicate how an innovative product, such as eggs from laying hens fed on a diet based on insect meal, records a significant consumers’ acceptability, with about 75% of respondents expressing a favorable opinion about insect-based animal feed. Previous studies recorded about 90% of the respondents having a positive attitude toward the use of insect meal as feed in fish farming [36].

However, not all consumers have the same level of readiness for adopting novel foods. The questionnaire investigated the actual willingness to buy and eat eggs from IFH to segment consumers. This approach led to the creation of four clusters, differing for the inclination to accept insect-based feed, food habits, environmentally friendly food choice, and level of neophobia. Participants’ segmentation showed cluster “ready” being the largest. Results are in agreement with a previous Italian study, where the majority of respondents stated that they were ready to accept insects in animal feed [30,37]. Results indicate that the consumption intention of the “ready” cluster toward eggs from insect-fed hens can be supported by the indication of product origin, especially if perceived as local. The “environmentalist” cluster, including participants favorable to choose IFH eggs due to their reduced environmental impact, is characterized by the lowest frequency of meat consumption. This indication, deriving from declared food habits responses, can be of use to plan an information strategy proposing eggs in alternative to meat, also taking into account that this cluster is the most inclined to reduce meat consumption to contribute to environmental sustainability. The “ready” and “environmentalist” clusters, sharing the highest acceptance of insects as feed, represent about two-thirds of the surveyed population. The “cautious” cluster is also in favor of the use of insect meal in hen feeding, although at a lower level. Conjoint analysis indicates how an area of the origin, particularly if local or at least domestic, may positively affect this cluster’s interest. To increase IFH eggs’ appeal by this cluster, producers and retailers should focus on providing information about the place of origin while emphasizing the benefit for the environment. Positive effects of information were reported on French consumers, in research on insect-fed trout, with informed consumers showing higher acceptance than the uninformed ones [38]. Questionnaire results also evidenced a cluster of “reluctant” participants, unwilling to buy IFH eggs and contrary to the use of insects as feed. This cluster was also characterized by the highest level of neophobia, indicating the negative effect of this factor on novel food acceptance. The recorded decrease in the acceptance of insect-fed animal products, correlated with an increase in neophobic attitude, indicates the importance of neophobia in influencing an open approach to food novelty, which corresponds to the results of recent studies [25,39,40,41]. The size of the “reluctant” cluster is about one-sixth of the participants, and is consistent with results of previous studies [36,42], and does not seem to affect the market potential of the proposed product. Other studies have indicated that the use of insects in foodstuffs and feed may scale up consumers’ risk perception [42], and adequate information on risks and benefits may influence positively “reluctant” views, widening the range of potential consumers.

Concerning the simulated commercial offer of IFH eggs, as expected, the presence of insect meal in hens feed negatively affected product evaluation from the “reluctant” cluster, which also expressed a negative evaluation for organic rearing while resulting in not being concerned about price. On the other hand, the other three clusters were positively influenced by a label indicating the “use of insects as a feed”; in addition, they shared a common interest in a low price product and a positive opinion of the organic rearing system.

One of the limitations of the study is the relatively low number of participants, as compared to the whole Italian population. Another limiting point is that the proposed product is not yet offered on the market; thus, the data resented and discussed should be validated once IFH eggs are available.

## 5. Conclusions

The main issue of this work was to investigate the readiness of Italian consumers to adopt eggs from insect-fed hens. Participants in an online survey expressed a high level of willingness to accept this new product. Nevertheless, since a small percentage of consumers is still skeptical, the gathered information on the obtained acceptance clusters, about price, rearing systems, egg attributes, environmental issues, and information about the origin can be of help to increase the acceptance of innovative production systems of eggs.

## Figures and Tables

**Table 1:** Attributes and levels presented in the survey.

Levels	Attributes
	Price
1	1.55 €
2	1.85 €
3	2.50 €
	Rearing system
1	Free-range
2	Barn
3	Organic
	Insects
1	Yes
2	No

**Table 2:** Description and distribution of the sample; 510 respondents.

Description	*n*	%
Total respondents	510	100%
Gender		
Female	245	48%
Male	259	51%
Undisclosed	6	1%
Education Background		
None/Primary/Lower secondary/Upper secondary school	108	21%
Degree	233	46%
Post degree (MSc; PhD)	165	32%
Undisclosed	4	1%
Place of residence		
Urban area	284	56%
Suburban area	106	20%
Rural area	115	23%
Undisclosed	5	1%
Income Level (€/year)		
<10,000	55	11%
Between 10,000 and 20,000	100	20%
>20,000	136	26%
>30,000	151	30%
>50,000	52	10%
Undisclosed	16	3%
Place of residence		

**Table 3:** Cluster obtained from answers to question on the willingness to buy and eat IFH eggs.

Cluster	Number of Respondents	Percentage
Ready	205	40.1
Environmentalist	123	24.3
Cautious	111	21.4
Reluctant	71	13.9
Total sample	510	100

**Table 4:** Neophobia mean score (FNS) and acceptance mean score calculated for total population and consumer clusters.

Attributes	Total Sample		Ready		Environmentalist		Cautious		Reluctant	
	Mean	SEM	Mean	SEM	Mean	SEM	Mean	SEM	Mean	SEM
FNS	29.51	0.45	27.91 b	0.70	28.01 b	0.87	29.49 b	0.89	36.73 a	1.13
Acc. Score	5.48	0.07	6.00 a	0.09	6.01 a	0.10	5.47 b	0.12	3.10 c	0.19

a, b, c: Means with different letters correspond to statistical differences among the clusters (*p* ≤ 0.05). SEM: standard error of the mean.

**Table 5:** Average consumption of selected food types, based on frequency (1: never, 2: once a week, 3: twice a week, 4: more than twice a week, 5: every day) for total population and consumer clusters.

Parameters	Total Sample		Ready		Environmentalist		Cautious		Reluctant	
	Mean	SEM	Mean	SEM	Mean	SEM	Mean	SEM	Mean	SEM
Meat	2.23	0.03	2.31 a	0.06	2.04 b	0.07	2.25 a	0.06	2.26 a	0.09
Eggs	2.43	0.04	2.52 ns	0.06	2.34 ns	0.08	2.36 ns	0.08	2.42 ns	0.10
Legumes	2.51	0.05	2.66 a	0.08	2.60 a	0.10	2.20 b	0.10	2.41 b	0.14
Vegetables	4.57	0.03	4.65 a	0.04	4.58 ab	0.07	4.52 ab	0.07	4.39 b	0.12
Fresh Fruit	2.57	0.07	2.64 ns	0.11	2.66 ns	0.13	2.41 ns	0.14	2.49 ns	0.17
Milk Products	3.46	0.05	3.46 ns	0.09	3.37 ns	0.12	3.60 ns	0.11	3.38 ns	0.14
Fish	2.22	0.04	2.34 ns	0.06	2.12 ns	0.08	2.15 ns	0.08	2.14 ns	0.10
Shell fruit	4.39	0.05	4.37 ns	0.070	4.48 ns	0.08	4.47 ns	0.09	4.20 ns	0.15
Cereals	3.65	0.07	3.77 ns	0.10	3.74 ns	0.14	3.51 ns	0.15	3.35 ns	0.19

a, b: Means with different letters correspond to statistical differences among the clusters (*p* ≤ 0.05), ns = not significant. SEM: standard error of the mean.

**Table 6:** Contingency tables reporting the percentage of individual sustainable food choices for each consumer cluster.

Cluster	Label *	LI-Brand ns	LI-Product ns	Local ns	No Import ns	Reduce Meat **	Organic *	Origin *	Seasonality ns
Ready	39.0	28.8	0.1	53.7	28.8	40.5	19.0	71.2	83.9
Environmentalist	43.9	34.1	10.6	60.2	26.8	58.5	30.9	74.8	82.1
Cautious	51.3	32.4	0.1	55.0	22.5	41.4	20.7	73.9	87.4
Reluctant	52.1	43.7	14.1	47.9	33.8	38.0	25.3	67.6	81.7
Total consumers	44.7	32.9	9.0	54.7	27.6	44.7	23.1	72.2	83.9

Results of Cochran’s Q test are shown next to each attribute: * indicates significant differences among samples at *p* < 0.10, ** indicates significant differences among samples at *p* < 0.05, whereas ns indicates no significant differences (*p* > 0.10).

**Table 7:** Importance of egg attributes for total population and consumer clusters.

Attributes	Total Sample		Ready		Environmentalist		Cautious		Reluctant	
	Mean	SEM	Mean	SEM	Mean	SEM	Mean	SEM	Mean	SEM
Size	3.26	0.076	3.18 a	0.116	3.11 a	0.166	3.41 a	0.153	3.54 a	0.218
Shell Color	2.77	0.074	2.65 ab	0.117	2.56 b	0.150	3.02 ab	0.149	3.08 a	0.211
Breeding	5.97	0.065	5.83 a	0.110	6.04 a	0.127	6.14 a	0.122	5.96 a	0.185
Yolk Color	4.06	0.086	3.95 bc	0.134	3.59 c	0.175	4.29 b	0.176	4.83 a	0.231
Feeding	5.01	0.088	4.73 b	0.146	5.02 ab	0.176	5.21 ab	0.166	5.46 a	0.232
Brand	3.88	0.085	3.72 b	0.134	3.66 b	0.175	3.96 b	0.166	4.59 a	0.234
Origin	5.64	0.072	5.45 b	0.121	5.47 b	0.153	5.91 a	0.128	6.08 a	0.168
Label	3.97	0.091	3.42 b	0.145	4.12 a	0.175	4.55 a	0.178	4.39 a	0.253

a, b, c: Means with different letters correspond to statistical differences among the clusters (*p* ≤ 0.05). SEM: standard error of the mean.

**Table 8:** Conjoint analysis: relative importance and utilities of total sample and by clusters.

Attribute	Attribute Level	Total	Ready	Environmentalist	Cautious	Reluctant
Price	Relative importance (%)	41.2	43.4	43.0	45.0	21.0
	1.55 €	0.380	0.407	0.461	0.407	0.389
	1.85 €	0.156	0.141	0.196	0.191	−0.611
	2.50 €	−0.536	−0.548	−0.657	−0.598	0.222
Rearing system	Relative importance (%)	33.4	34.2	34.0	34.0	31.0
	Free-range	−0.319	−0.307	−0.445	−0.296	0.722
	Barn	0.051	0.086	0.024	0.021	0.056
	Organic	0.268	0.220	0.422	0.275	−0.778
Insects	Relative importance (%)	25.4	22.4	23.0	21.0	48.0
	Yes	0.020	0.195	0.219	0.122	−1.167
	No	−0.020	−0.195	−0.219	−0.122	1.167

## Data Availability

The data presented in this study are available on request from the corresponding author.
